# Kernel Risk-Sensitive Mean *p*-Power Error Algorithms for Robust Learning

**DOI:** 10.3390/e21060588

**Published:** 2019-06-13

**Authors:** Tao Zhang, Shiyuan Wang, Haonan Zhang, Kui Xiong, Lin Wang

**Affiliations:** 1 College of Electronic and Information Engineering, Southwest University, Chongqing 400715, China; 2 Chongqing Key Laboratory of Nonlinear Circuits and Intelligent Information Processing, Chongqing 400715, China

**Keywords:** correntropic, quantized, kernel risk-sensitive mean p-power error, recursive, kernel adaptive filters

## Abstract

As a nonlinear similarity measure defined in the reproducing kernel Hilbert space (RKHS), the correntropic loss (C-Loss) has been widely applied in robust learning and signal processing. However, the highly non-convex nature of C-Loss results in performance degradation. To address this issue, a convex kernel risk-sensitive loss (KRL) is proposed to measure the similarity in RKHS, which is the risk-sensitive loss defined as the expectation of an exponential function of the squared estimation error. In this paper, a novel nonlinear similarity measure, namely kernel risk-sensitive mean *p*-power error (KRP), is proposed by combining the mean *p*-power error into the KRL, which is a generalization of the KRL measure. The KRP with p=2 reduces to the KRL, and can outperform the KRL when an appropriate *p* is configured in robust learning. Some properties of KRP are presented for discussion. To improve the robustness of the kernel recursive least squares algorithm (KRLS) and reduce its network size, two robust recursive kernel adaptive filters, namely recursive minimum kernel risk-sensitive mean *p*-power error algorithm (RMKRP) and its quantized RMKRP (QRMKRP), are proposed in the RKHS under the minimum kernel risk-sensitive mean *p*-power error (MKRP) criterion, respectively. Monte Carlo simulations are conducted to confirm the superiorities of the proposed RMKRP and its quantized version.

## 1. Introduction

Online kernel-based learning is to extend the kernel methods to online settings where the data arrives sequentially, which has been widely applied in signal processing thanks to its excellent performance in addressing nonlinear issues [1]. The development of kernel methods is of great significance for practical applications. In kernel methods, the input data are transformed from the original space into the reproducing kernel Hilbert space (RKHS) using the kernel trick [2]. As the representative of the kernel methods, kernel adaptive filters (KAFs) provide an effective way to transform a nonlinear problem into a linear one, which have been widely introduced in system identification and time-series prediction [3,4,5]. Generally, KAFs are designed for Gaussian and non-Gaussian noises from the aspect of cost function, respectively.

For Gaussian noises, the second-order similarity measures of errors are generally used as a cost function of KAFs to achieve desirable filtering accuracy. Therefore, in the Gaussian noise environment, KAFs based on the second-order similarity measures of errors are mainly divided into three categories, i.e., the kernel least mean square (KLMS) algorithm [6], the kernel affine projection algorithm (KAPA) [7], and the kernel recursive least square algorithm (KRLS) [8]. However, the network size of KAFs increases linearly with the length of training, leading to large computational and storage burdens. To curb this structure growth, many sparsification methods are required, such as the surprise criterion (SC) [9], novelty criterion (NC) [10], coherence criterion [11], and approximate linear dependency (ALD) criterion [8]. However, these sparsification methods only discard the redundant data, leading to reduction of filtering accuracy. Unlike the aforementioned sparsification methods, the vector quantization (VQ) utilizes the redundant data to update the weights for accuracy improvement. The VQ is combined into KAFs to generate quantized KAFs, e.g., the quantized kernel least mean square algorithm (QKLMS) [12] and quantized kernel recursive least squares algorithm (QKRLS) [13].

However, the second-order similarity measures used in the aforementioned algorithms merely contain the second order statistics of errors, which cannot address non-Gaussian noises or outliers, efficiently [14]. Thus, it is very important to design a cost function beyond the second-order statistics of errors for combating non-Gaussian noises. The non-second order similarity measures can be divided into three categories, i.e., the mean *p*-power error (MPE) criterion [15], information theoretic learning (ITL) [14], and risk-sensitive loss (RL) based criteria [16,17]. The MPE criterion based on the *p*th absolute moment of the error can deal with non-Gaussian data with a proper *p*-value, efficiently. In general, MPE is robust to large outliers when p<2 [15], generating robust adaptive filters [15], e.g., the kernel least mean *p*-power (KLMP) algorithm [18] and the kernel recursive least mean *p*-power (KRLP) algorithm [18]. ITL can incorporate the complete distribution of errors into the learning process, resulting in the improvement of filtering precision and robustness to outliers. The most widely used ITL criterion is the maximum correntropy criterion (MCC) [19,20,21,22,23,24]. As a local similarity measure defined as a generalized correlation in the RKHS, the correntropy used in MCC can leverage higher order statistics of data to combat outliers [25]. However, the performance surface of the correntropic loss (C-Loss) is highly non-convex, which may lead to poor convergence performance. In the RL-based criteria, e.g., minimum risk-sensitive loss [16] and minimum kernel risk-sensitive loss (MKRL) [17,26], the risk-sensitive loss in the RKHS is convex extremely, which is more efficient for combating non-Gaussian noises or outliers than MCC [17,26]. However, since the MKRL uses the stochastic gradient descent (SGD) method to update its weights, the desirable filtering performance cannot be achieved for some complex nonlinear issues. The recursive update rules with excellent tracking ability can improve the filtering performance of adaptive filtering algorithms [21]. For example, KRLS based on the recursive update rule can improve the filtering performance of KLMS based on the SGD, significantly. To the best of our knowledge, however, it has not yet been proposed to design a recursive MKRL algorithm for desirable filtering performance in the RKHS by a recursive update rule.

In this paper, to inherit the advantages of both KRL and MPE for robustness improvement, we propose the risk-sensitive mean *p*-power error (RP) defined as the expectation of an exponential function of the *p*th absolute moment of the estimation error, and its kernel RP (KRP). The KRP can outperform the KRL by setting an appropriate *p*-value for robust learning, and the KRP with p=2 reduces to the KRL. The proposed KRP criterion is used to derive a novel recursive minimum kernel risk-sensitive mean *p*-power error (RMKRP) algorithm for desirable filtering performance by combining the weighted output information. Furthermore, to curb the growth of network size in the RMKRP, the VQ is combined into RMKRP to generate quantized RMKRP (QRMKRP).

The rest of this paper is organized as follows. In Section 2, we define the KRP, and give some basic properties. The KRP criterion is derived to develop a recursive adaptive algorithm by combining the weighted output information, called RMKRP algorithm in Section 3. To further reduce the network size of RMKRP, the vector quantization method is applied in RMKRP, thus generating the quantized RMKRP (QRMKRP) in Section 3. In Section 4, Monte Carlo simulations are conducted to validate the superiorities of the proposed algorithms in nonlinear examples. The conclusion is summarized in Section 5.

## 2. Kernel Risk-Sensitive Mean *p*-Power Error

### 2.1. Definition

According to [17], the risk-sensitive loss is defined in RKHS, called the kernel risk-sensitive loss (KRL). Given two arbitrary scalar random variables *X* and *Y*, where X,Y∈R, the KRL is defined by

(1) $$\begin{array}{cc} L_{\lambda }\left(X,Y\right) & =\frac{1}{\lambda }E\left[\exp\left(\lambda \left(\frac{1}{2}{∥\varphi (X)-\varphi (Y)∥}_{F}^{2}\right)\right)\right]\\ & =\frac{1}{\lambda }E\left[\exp\left(\lambda \left(\frac{1}{2}{\langle \varphi (X)-\varphi (Y),\varphi (X)-\varphi (Y)\rangle }_{F}\right)\right)\right]\\ & =\frac{1}{\lambda }E\left[\exp\left(\lambda \left(\frac{1}{2}\left({\langle \varphi (X),\varphi (X)\rangle }_{F}+{\langle \varphi (Y),\varphi (Y)\rangle }_{F}-2{\langle \varphi (X),\varphi (Y)\rangle }_{F}\right)\right)\right)\right]\\ & =\frac{1}{\lambda }E\left[\exp\left(\lambda \left(1-\kappa_{\sigma }\left(X-Y\right)\right)\right)\right]\\ & =\frac{1}{\lambda }\int \exp(\lambda \left(1-\kappa_{\sigma }\left(X-Y\right)\right))dF_{XY}\left(x,y\right), \end{array}$$

where λ>0 is a risk-sensitive scalar parameter; φ(X)=κ(X,.) is a nonlinear mapping induced by a Mercer kernel $\kappa_{\sigma }\left(.\right)$, which transforms the data from the original space into the RKHS F equipped with an inner product ${\langle .,.\rangle }_{F}$ satisfying ${\langle \varphi (X),\varphi (Y)\rangle }_{F}=\varphi^{T}\left(X\right)\varphi \left(Y\right)=\kappa_{\sigma }\left(X-Y\right)$; E denotes the mathematical expectation; ${∥.∥}_{F}$ denotes the norm in RKHS F; $F_{XY}\left(x,y\right)$ denotes the joint distribution function of (X,Y). A shift-invariant Gaussian kernel $\kappa_{\sigma }\left(.\right)$ with bandwidth σ is given as follows:

(2) $$\begin{array}{c} \kappa_{\sigma }\left(x,y\right)=\kappa_{\sigma }\left(x-y\right)=\exp\left(-\frac{{\left(x-y\right)}^{2}}{2\sigma^{2}}\right). \end{array}$$

However, the joint distribution of (X,Y) is usually unknown, and only *N* samples ${\left\{x\left(i\right),y\left(i\right)\right\}}_{i=1}^{N}$ are available. Hence, the nonparametric estimate of $L_{\lambda }\left(X,Y\right)$ is obtained by applying the Parzen windows [19] as ${\hat{L}}_{\lambda }\left(X,Y\right)=\frac{1}{N\lambda }\sum_{i=1}^{N}\exp(\lambda \left(1-\kappa_{\sigma }\left(x\left(i\right)-y\left(i\right)\right)\right))$. Note that the inner product in the RKHS for the same input is calculated by using kernel trick and (2), i.e, $\varphi^{T}\left(X\right)\varphi \left(X\right)=\exp\left(-\frac{{\left(X-X\right)}^{2}}{2\sigma^{2}}\right)=1$.

In this paper, we define a new non-second order similarity measure in the RKHS, i.e., the kernel risk-sensitive mean *p*-power error (KRP) loss. Given two random variables *X* and *Y*, the KRP loss is defined by

(3) $$\begin{array}{cc} L_{\lambda ,p}\left(X,Y\right) & =\frac{1}{\lambda }E\left[\exp\left(\lambda 2^{-p/2}\left({∥\varphi (X)-\varphi (Y)∥}_{F}^{p}\right)\right)\right]\\ & =\frac{1}{\lambda }E\left[\exp\left(\lambda 2^{-p/2}{\left({∥\varphi (X)-\varphi (Y)∥}_{F}^{2}\right)}^{p/2}\right)\right]\\ & =\frac{1}{\lambda }E\left[\exp\left(\lambda 2^{-p/2}{\left(2-2\kappa_{\sigma }\left(X-Y\right)\right)}^{p/2}\right)\right]\\ & =\frac{1}{\lambda }E\left[\exp\left(\lambda {\left(1-\kappa_{\sigma }\left(X-Y\right)\right)}^{p/2}\right)\right]\\ & =\frac{1}{\lambda }\int \exp\left(\lambda {\left(1-\kappa_{\sigma }\left(X-Y\right)\right)}^{p/2}\right)dF_{X,Y}\left(x,y\right), \end{array}$$

where p>0 is the power parameter. Note that the KRL can be regarded as a special case of the KRP with p=2.

However, the joint distribution of *X* and *Y* is usually unknown in practice. Hence, the empirical KRP is defined as follows:

(4) $$\begin{array}{c} {\hat{L}}_{\lambda ,p}\left(X,Y\right)=\frac{1}{N\lambda }\sum_{i=1}^{N}\exp(\lambda {\left(1-\kappa_{\sigma }\left(x\left(i\right)-y\left(i\right)\right)\right)}^{p/2}), \end{array}$$

where ${\left\{x\left(i\right),y\left(i\right)\right\}}_{i=1}^{N}$ denotes the available finite number of samples. The empirical KRP can be regarded as a distance between both $X={\left[x\left(1\right),x\left(2\right),\ldots ,x\left(N\right)\right]}^{T}$ and $Y={\left[y\left(1\right),y\left(2\right),\ldots ,y\left(N\right)\right]}^{T}$.

### 2.2. Properties

In the following, we give some important properties of the proposed KRP.

**Property** **1.** *$L_{\lambda ,p}\left(X,Y\right)$ is symmetric that is $L_{\lambda ,p}\left(X,Y\right)=L_{\lambda ,p}\left(Y,X\right)$.*

**Proof.**  Straightforward since $\kappa_{\sigma }\left(X-Y\right)=\kappa_{\sigma }\left(Y-X\right)$. □

**Property** **2.** *$L_{\lambda ,p}\left(X,Y\right)$ is positive and bounded, i.e., $\frac{1}{\lambda }\leq L_{\lambda ,p}\left(X,Y\right)\leq \frac{1}{\lambda }\exp\left(\lambda \right)$, and reaches its minimum if X=Y.*

**Proof.**  Straightforward since $0<\kappa_{\sigma }\left(X-Y\right)\leq 1$, and $\kappa_{\sigma }\left(X-Y\right)=1$ if X=Y. □

**Property** **3.** *As λ is small enough, it holds that $L_{\lambda ,p}\left(X,Y\right)\approx \frac{1}{\lambda }+E\left[{\left(1-\kappa_{\sigma }\left(X-Y\right)\right)}^{p/2}\right]$.*

**Proof.**  For a small enough λ, we have $\lambda {\left(1-\kappa_{\sigma }\left(X-Y\right)\right)}^{p/2}\to 0$, i.e.,

(5) $$\begin{array}{c} \exp\left(\lambda {\left(1-\kappa_{\sigma }\left(X-Y\right)\right)}^{p/2}\right)\approx 1+\lambda {\left(1-\kappa_{\sigma }\left(X-Y\right)\right)}^{p/2}. \end{array}$$

Therefore, we can obtain

(6) $$\begin{array}{cc} L_{\lambda ,p}\left(X,Y\right) & =\frac{1}{\lambda }E\left[\exp\left(\lambda {\left(1-\kappa_{\sigma }\left(X-Y\right)\right)}^{p/2}\right)\right]\\ & \overset{\left(5\right)}{\approx }\frac{1}{\lambda }E\left[1+\lambda {\left(1-\kappa_{\sigma }\left(X-Y\right)\right)}^{p/2}\right]\\ & =\frac{1}{\lambda }+E\left[{\left(1-\kappa_{\sigma }\left(X-Y\right)\right)}^{p/2}\right]. \end{array}$$

 □

**Property** **4.** *As σ is large enough, it holds that $L_{\lambda ,p}\left(X,Y\right)\approx \frac{1}{\lambda }+{\left(2\sigma^{2}\right)}^{-p/2}E[|X-Y{|}^{p}]$.*

**Proof.**  Since exp(x) is approximated by 1+x for a small enough *x*, for the case of large enough σ, i.e., $\frac{{\left(X-Y\right)}^{2}}{2\sigma^{2}}\to 0$. Thus, we can obtain the approximation as

(7) $$\begin{array}{c} \exp\left(-\frac{{\left(X-Y\right)}^{2}}{2\sigma^{2}}\right)\approx 1-\frac{{\left(X-Y\right)}^{2}}{2\sigma^{2}}. \end{array}$$

Similarly, when $\lambda {\left(\frac{{\left(X-Y\right)}^{2}}{2\sigma^{2}}\right)}^{p/2}\to 0$ for large enough σ, we can also obtain the approximation as

(8) $$\begin{array}{c} \exp\left(\lambda {\left(\frac{{\left(X-Y\right)}^{2}}{2\sigma^{2}}\right)}^{p/2}\right)\approx 1+\lambda {\left(\frac{{\left(X-Y\right)}^{2}}{2\sigma^{2}}\right)}^{p/2}. \end{array}$$

According to (7) and (8), we have

(9) $$\begin{array}{cc} L_{\lambda ,p}\left(X,Y\right) & =\frac{1}{\lambda }E\left[\exp\left(\lambda {\left(1-\exp\left(-\frac{{\left(X-Y\right)}^{2}}{2\sigma^{2}}\right)\right)}^{p/2}\right)\right]\\ & \overset{\left(7\right)}{\approx }\frac{1}{\lambda }E\left[\exp\left(\lambda {\left(\frac{{\left(X-Y\right)}^{2}}{2\sigma^{2}}\right)}^{p/2}\right)\right]\\ & \overset{\left(8\right)}{\approx }\frac{1}{\lambda }E\left[1+\lambda {\left(\frac{{\left(X-Y\right)}^{2}}{2\sigma^{2}}\right)}^{p/2}\right]\\ & =\frac{1}{\lambda }+{\left(2\sigma^{2}\right)}^{-p/2}E\left[{\left|X-Y\right|}^{p}\right]. \end{array}$$

 □

**Remark** **1.** *According to Properties *3* and *4*, the KRP is, approximately, equivalent to the KMPE [27] as λ is small enough, and equivalent to the MPE [15] as σ is large enough. Thus, the KMPE and MPE can be viewed as two extreme cases of the KRP.*

**Property** **5.** *As p is small enough, it holds that $L_{\lambda ,p}\left(X,Y\right)\approx \frac{1}{\lambda }\exp\left(\lambda \left(1+\left(p/2)E[\log(1-\kappa_{\sigma }\left(X-Y\right)\right)\right]\right))\approx \frac{1}{\lambda }\exp\left(\lambda \right)$.*

**Proof.**  Property 5 holds because of $(1-\kappa_{\sigma }{\left(X-Y\right))}^{p/2}\approx 1+\left(p/2)E[\log(1-\kappa_{\sigma }\left(X-Y\right)\right)]\approx 1$. □

**Property** **6.** *Let $e=X-Y={\left[e\left(1\right),e\left(2\right),\ldots ,e\left(N\right)\right]}^{T}$, where e(i)=x(i)−y(i). The empirical KRP ${\hat{L}}_{\lambda ,p}\left(X,Y\right)$ as a function of e is convex at any point satisfying ${∥e∥}_{\infty }=\max_{i=1,2,\ldots ,N}\left|e(i)\right|\leq \sigma$ and p≥2. When ${∥e∥}_{\infty }>\sigma$, the empirical KRP ${\hat{L}}_{\lambda ,p}\left(X,Y\right)$ is also convex if the risk-sensitive parameter λ>0 and power parameter $p\geq \max_{i=1,2,\ldots ,N}\left\{\frac{2\left(e^{2}\left(i\right)-\sigma^{2}\right)\left(1-\kappa_{\sigma }\left(e\left(i\right)\right)\right)}{e^{2}\left(i\right)\kappa_{\sigma }\left(e\left(i\right)\right)}+2\right\}$.*

**Proof.**  Since ${\hat{L}}_{\lambda ,p}\left(X,Y\right)=\frac{1}{N\lambda }\sum_{i=1}^{N}\exp(\lambda {\left(1-\kappa_{\sigma }\left(e\left(i\right)\right)\right)}^{p/2})$, the Hessian matrix of ${\hat{L}}_{\lambda ,p}\left(X,Y\right)$ with respect to e can be derived as

(10) $$\begin{array}{c} H_{{\hat{L}}_{\lambda ,p}\left(X,Y\right)}\left(e\right)=\left[\frac{\partial^{2}{\hat{L}}_{\lambda ,p}\left(X,Y\right)}{\partial e(i)\partial e(j)}\right]=\mathrm{diag}\left[\gamma_{1},\gamma_{2},\ldots ,\gamma_{N}\right], \end{array}$$

where

(11) $$\begin{array}{cc} \gamma_{i}= & \zeta_{i}\left(\frac{p\lambda }{2\sigma^{2}}{\left(1-\kappa_{\sigma }\left(e\left(i\right)\right)\right)}^{(p-2)/2}\exp\left(-\frac{e^{2}\left(i\right)}{2\sigma^{2}}\right)e^{2}\left(i\right)+1\right.\\ & +\left.\frac{p-2}{2}{\left(1-\kappa_{\sigma }\left(e\left(i\right)\right)\right)}^{-1}\exp\left(-\frac{e^{2}\left(i\right)}{2\sigma^{2}}\right)\frac{e^{2}\left(i\right)}{\sigma^{2}}-\frac{e^{2}\left(i\right)}{\sigma^{2}}\right), \end{array}$$

with $\zeta_{i}=\frac{p}{2N\sigma^{2}}\exp\left(\lambda {\left(1-\kappa_{\sigma }\left(e\left(i\right)\right)\right)}^{p/2}\right){\left(1-\kappa_{\sigma }\left(e\left(i\right)\right)\right)}^{(p-2)/2}\exp\left(-\frac{e^{2}\left(i\right)}{2\sigma^{2}}\right),i=1,2,\ldots ,N$. When p≥2, we have $H_{{\hat{L}}_{\lambda ,p}\left(X,Y\right)}\left(e\right)>0$ if $\max_{i=1,2,\ldots ,N}\left|e(i)\right|\leq \sigma$. From (11), if |e(i)|≤σ and p≥2, or |e(i)|>σ and $p\geq [2\left(e^{2}\left(i\right)-\sigma^{2}\right)\left(1-\kappa_{\sigma }\left(e\left(i\right)\right)\right)/(e^{2}\left(i\right)\kappa_{\sigma }\left(e\left(i\right)\right))]+2$, we have $\zeta_{i}\geq 0$. Therefore, we have $H_{{\hat{L}}_{\lambda ,p}\left(X,Y\right)}\left(e\right)\geq 0$ if

(12) $$\begin{array}{c} p\left(\lambda {\left(1-\kappa_{\sigma }\left(e\left(i\right)\right)\right)}^{p/2}+1\right)\geq \max_{i=1,2,\ldots ,N}\left\{\frac{2\left(e^{2}\left(i\right)-\sigma^{2}\right)\left(1-\kappa_{\sigma }\left(e\left(i\right)\right)\right)}{e^{2}\left(i\right)\kappa_{\sigma }\left(e\left(i\right)\right)}+2\right\}, \end{array}$$

where $(\lambda {\left(1-\kappa_{\sigma }\left(e\left(i\right)\right)\right)}^{p/2}+1)\geq 1$. Thus, we have $p\geq \max_{i=1,2,\ldots ,N}\left\{\frac{2\left(e^{2}\left(i\right)-\sigma^{2}\right)\left(1-\kappa_{\sigma }\left(e\left(i\right)\right)\right)}{e^{2}\left(i\right)\kappa_{\sigma }\left(e\left(i\right)\right)}+2\right\}$. □

**Remark** **2.** *According to Property *6*, the empirical KRP as a function of e is convex at any point satisfying ${∥e∥}_{\infty }\leq \sigma$ and p≥2. For the case ${∥e∥}_{\infty }>\sigma$, the empirical KRP can still be convex at a point if the risk-sensitive parameter λ>0 and power parameter $p\geq \max_{i=1,2,\ldots ,N}\left\{\frac{2\left(e^{2}\left(i\right)-\sigma^{2}\right)\left(1-\kappa_{\sigma }\left(e\left(i\right)\right)\right)}{e^{2}\left(i\right)\kappa_{\sigma }\left(e\left(i\right)\right)}+2\right\}$.*

**Property** **7.** *As σ→∞ or x(i)→0, i=1,2,…,N, it holds that*

(13) $$\begin{array}{c} {\hat{L}}_{\lambda ,p}\left(X,0\right)\approx \frac{1}{\lambda }+\frac{1}{N{\left(\sqrt{2}\sigma \right)}^{p}}{∥X∥}_{p}^{p}, \end{array}$$

*where 0 denotes an N-dimensional zero vector.*

**Proof.**  (14) $$\begin{array}{cc} {\hat{L}}_{\lambda ,p}\left(X,0\right) & =\frac{1}{N\lambda }\sum_{i=1}^{N}\exp(\lambda {\left(1-\kappa_{\sigma }\left(x\left(i\right)\right)\right)}^{p/2})\approx \frac{1}{N\lambda }\sum_{i=1}^{N}\exp\left(\lambda {\left(1-\left(1-\frac{x^{2}\left(i\right)}{2\sigma^{2}}\right)\right)}^{p/2}\right)\\ & =\frac{1}{N\lambda }\sum_{i=1}^{N}\exp\left(\lambda {\left(\frac{x^{2}\left(i\right)}{2\sigma^{2}}\right)}^{p/2}\right)\approx \frac{1}{N\lambda }\sum_{i=1}^{N}\left[1+\lambda {\left(\frac{x^{2}\left(i\right)}{2\sigma^{2}}\right)}^{p/2}\right]\\ & =\frac{1}{\lambda }+\frac{1}{N{\left(\sqrt{2}\sigma \right)}^{p}}\sum_{i=1}^{N}|x\left(i\right){|}^{p}=\frac{1}{\lambda }+\frac{1}{N{\left(\sqrt{2}\sigma \right)}^{p}}{∥X∥}_{p}^{p}. \end{array}$$

 □

**Remark** **3.** *According to Property *7*, the empirical KRP ${\hat{L}}_{\lambda ,p}\left(X,0\right)$ behaves like an $L_{p}$ norm of X when kernel bandwidth σ is large enough.*

## 3. Application to Adaptive Filtering

In this section, to combat non-Gaussian noises, two recursive robust adaptive algorithms under the proposed KRP criterion are proposed in the RKHS using the kernel trick and vector quantization technique, respectively.

### 3.1. RMKRP

The recursive strategy is introduced into the KRP loss function, namely the recursive minimum kernel risk-sensitive mean *p*-power error (RMKRP) algorithm. The offline solution to minimum of the KRP loss is first obtained. Based on the obtained offline solution, the recursive solution or online solution to minimum of the KRP loss is then derived using some matrix operations, which generates the RMKRP algorithm. The details of RMKRP are shown as follows.

**Figure 1:**
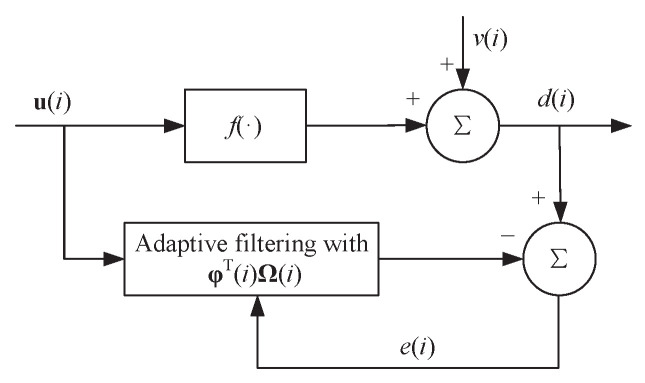
Block diagram of adaptive filtering.

Consider the prediction of a continuous input-output model f:U→R based on adaptive filtering shown in Figure 1, where $u\left(i\right)\in U\subset R^{D}$ is the *i*th *D*-dimensional input vector, d(i)∈R is the *i*th scalar desired output contaminated by a noise v(i), i.e., d(i)=f(u(i))+v(i). A sequence of training samples ${\left\{u(j),d(j)\right\}}_{j=1}^{i}$ is used to perform the prediction of f(·) in an adaptive filter. The nonlinear mapping φ(u(j)) of input u(j) is denoted by φ(j) for simplicity. Hence, in the RKHS F, the training samples are changed to $\left\{\Phi (i),d(i)\right\}$, where the desired output vector is $d\left(i\right)={\left[d\left(1\right),d\left(2\right),\ldots ,d\left(i\right)\right]}^{T}$ and the input kernel mapping matrix is Φ(i)=[φ(1),φ(2),…,φ(i)]. The prediction denoted by $\hat{f}\left(\cdot \right)$ in the RKHS is therefore given as $\hat{f}\left(\cdot \right)=\varphi^{T}\left(\cdot \right)\Omega$, where Ω∈F is the weight vector in a high dimensional feature space F.

An exponentially-weighted loss function is used here to put more emphasis on recent data and to de-emphasize data on the remote past [28]. When $\left\{\Phi (i),d(i)\right\}$ are available, the weight vector Ω(i) is obtained as the offline solution to minimizing the following weighted cost function:

(15) $$\begin{array}{c} J\left(\Omega \left(i\right)\right)=\sum_{j=1}^{i}\rho^{i-j}\frac{1}{\lambda }\exp\left(\lambda {\left(z(j)\right)}^{\frac{p}{2}}\right)+\frac{1}{2}\rho^{i}\zeta {∥\Omega (i)∥}^{2}, \end{array}$$

where ρ denotes the forgetting factor in the interval [0,1], ζ is the regularization factor, $z\left(j\right)=1-\exp\left(-\frac{e^{2}\left(j\right)}{2\sigma^{2}}\right)$, and $e\left(j\right)=d\left(j\right)-\varphi^{T}\left(j\right)\Omega \left(i\right)$ denotes the *j*th estimate error. The second term is a norm penalizing term, which is to guarantee the existence of the inverse of the input data autocorrelation matrix especially during the initial update stages. In addition, the regularization term is weighted by ρ, which deemphasizes regularization as time progresses. According to Property 6, the empirical KRP as a function of e is convex at any point satisfying $\max_{j=1,2,\ldots ,i}\left|e(j)\right|\leq \sigma$, λ>0, and p≥2. To obtain the minimum of (15), its gradient is calculated, i.e.,

(16) $$\begin{array}{cc} \frac{\partial J(\Omega (i))}{\partial \Omega (i)}= & -\frac{p}{2\sigma^{2}}\sum_{j=1}^{i}\varphi \left(j\right)\rho^{i-j}\exp\left(\lambda {\left(z(j)\right)}^{\frac{p}{2}}\right){\left(z(j)\right)}^{\frac{p-2}{2}}\left(d\left(j\right)-\varphi^{T}\left(j\right)\Omega \left(i\right)\right)\left(1-z\left(j\right)\right)+\rho^{i}\zeta \Omega \left(i\right)\\ = & -\frac{p}{2\sigma^{2}}\sum_{j=1}^{i}\varphi \left(j\right)\rho^{i-j}\exp\left(\lambda {\left(z(j)\right)}^{\frac{p}{2}}\right){\left(z(j)\right)}^{\frac{p-2}{2}}\left(1-z\left(j\right)\right)d\left(j\right)\\ & +\frac{p}{2\sigma^{2}}\sum_{j=1}^{i}\varphi \left(j\right)\rho^{i-j}\exp\left(\lambda {\left(z(j)\right)}^{\frac{p}{2}}\right){\left(z(j)\right)}^{\frac{p-2}{2}}\left(1-z\left(j\right)\right)\varphi^{T}\left(j\right)\Omega \left(i\right)+\rho^{i}\zeta \Omega \left(i\right). \end{array}$$

Setting (16) to zero, i.e., $\frac{\partial J(\Omega )}{\partial \Omega }=0$, we can obtain the offline solution to minimum of (15) as follows:

(17) $$\begin{array}{c} \Omega \left(i\right)={\left(\Phi \left(i\right)H\left(i\right)\Phi^{T}\left(i\right)+\rho^{i}\zeta 2\sigma^{2}/pI\right)}^{-1}\Phi \left(i\right)H\left(i\right)d\left(i\right), \end{array}$$

where $H\left(i\right)=\mathrm{diag}[H_{1}\left(i\right),H_{2}\left(i\right),\ldots ,H_{i}\left(i\right)]$ with $H_{j}\left(i\right)=\rho^{i-j}\exp\left(\lambda {\left(z(j)\right)}^{\frac{p}{2}}\right){\left(z(j)\right)}^{\frac{p-2}{2}}\left(1-z\left(j\right)\right)$, j=1,2,…,i, and I denotes an identity matrix with an appropriate dimension.

To obtain an efficient recursive solution to the minimum of (15), a Mercer kernel is used to construct the RKHS. Here, the Gaussian kernel is used as a Mercer kernel, which is denoted as $\kappa_{\sigma_{1}}\left(.\right)$ with $\sigma_{1}$ being the kernel width. The inner product in the RKHS can be calculated by using the kernel trick [28], i.e., $\kappa_{\sigma_{1}}\left(u\left(i\right),u\left(j\right)\right)=\kappa_{\sigma_{1}}\left(u\left(i\right)-u\left(j\right)\right)=\varphi^{T}\left(u\left(i\right)\right)\varphi \left(u\left(j\right)\right)=\varphi^{T}\left(i\right)\varphi \left(j\right)$, efficiently, which can avoid the direct calculation of nonlinear mapping φ(·).

Since the matrix inversion lemma [28] is described by ${\left(A+\mathrm{BCD}\right)}^{-1}=A^{-1}-A^{-1}B{\left(C^{-1}+DA^{-1}B\right)}^{-1}DA^{-1}$, by letting $A=\rho^{i}\zeta 2\sigma^{2}/pI$, B=Φ(i), C=H(i), and $D=\Phi^{T}\left(i\right)$, we rewrite (17) as

(18) $$\begin{array}{c} {\left(\Phi \left(i\right)H\left(i\right)\Phi^{T}\left(i\right)+\rho^{i}\zeta 2\sigma^{2}/pI\right)}^{-1}\Phi \left(i\right)H\left(i\right)=\Phi \left(i\right){\left(\Phi^{T}\left(i\right)\Phi \left(i\right)+\rho^{i}\zeta 2\sigma^{2}/pH{\left(i\right)}^{-1}\right)}^{-1}. \end{array}$$

Substituting (18) into (17) yields

(19) $$\begin{array}{c} \Omega \left(i\right)=\Phi \left(i\right){\left(\Phi^{T}\left(i\right)\Phi \left(i\right)+\rho^{i}\zeta 2\sigma^{2}/pH{\left(i\right)}^{-1}\right)}^{-1}d\left(i\right). \end{array}$$

Note that $\Phi^{T}\left(i\right)\Phi \left(i\right)$ in (19) can be computed by the kernel trick, efficiently. The weight vector Ω(i) is therefore described explicitly as a linear combination of the input data in the RKHS, i.e.,

(20) $$\begin{array}{c} \Omega (i)=\Phi (i)\alpha (i), \end{array}$$

where α(i) denotes the coefficients vector.

It can be seen from (20) that the recursive form of Ω(i) is changed to that of α(i). Hence, in the following, the key for finding a recursive solution to the minimum of (15) is to obtain the recursive form of α(i).

The coefficients vector α(i) is calculated using the kernel trick as

(21) $$\begin{array}{c} \alpha \left(i\right)={\left(\Phi^{T}\left(i\right)\Phi \left(i\right)+\rho^{i}\zeta 2\sigma^{2}/pH{\left(i\right)}^{-1}\right)}^{-1}d\left(i\right). \end{array}$$

For simplicity, we obtain the update form of α(i) indirectly by defining Λ(i) as

(22) $$\begin{array}{c} \Lambda \left(i\right)={\left(\Phi^{T}\left(i\right)\Phi \left(i\right)+\rho^{i}\zeta 2\sigma^{2}/pH{\left(i\right)}^{-1}\right)}^{-1}, \end{array}$$

where Φ(i)={Φ(i−1),φ(i)}. Then, the update form of Λ(i) can be further obtained

(23) $$\begin{array}{c} \Lambda \left(i\right)=\left[\begin{array}{c} \Phi^{T}\left(i-1\right)\Phi \left(i-1\right)+\rho^{i}\zeta 2\sigma^{2}/pH{\left(i-1\right)}^{-1}\\ \varphi^{T}\left(i\right)\Phi \left(i-1\right) \end{array}\right.{\left.\begin{array}{c} \Phi^{T}\left(i-1\right)\varphi \left(i\right)\\ \varphi^{T}\left(i\right)\varphi \left(i\right)+\rho^{i}\zeta 2\sigma^{2}/p\nu \left(i\right) \end{array}\right]}^{-1}, \end{array}$$

where $\nu \left(i\right)={\left(\exp\left(\lambda {\left(z(i)\right)}^{\frac{p}{2}}\right){\left(z(i)\right)}^{\frac{p-2}{2}}\left(1-z\left(i\right)\right)\right)}^{-1}$. By using some matrix operations, we further simplify (23) as

(24) $$\begin{array}{c} \Lambda {\left(i\right)}^{-1}=\left[\begin{array}{c} \Lambda {\left(i-1\right)}^{-1}\\ \xi^{T}\left(i\right) \end{array}\right.\left.\begin{array}{c} \xi (i)\\ \rho^{i}\zeta 2\sigma^{2}/p\nu \left(i\right)+\varphi^{T}\left(i\right)\varphi \left(i\right) \end{array}\right], \end{array}$$

where $\xi \left(i\right)=\Phi^{T}\left(i-1\right)\varphi \left(i\right)$. By using the following block matrix inversion identity [18,21,28]

(25) $$\begin{array}{c} \left[\begin{array}{c} A\\ C \end{array}\right.{\left.\begin{array}{c} B\\ D \end{array}\right]}^{-1}=\left[\begin{array}{c} {\left(A-BD^{-1}C\right)}^{-1}\\ -D^{-1}C{\left(A-BD^{-1}C\right)}^{-1} \end{array}\right.\left.\begin{array}{c} -A^{-1}B{\left(D-CA^{-1}B\right)}^{-1}\\ {\left(D-CA^{-1}B\right)}^{-1} \end{array}\right], \end{array}$$

then, we can obtain the update equation for the inverse of the growing matrix in (24) as

(26) $$\begin{array}{c} \Lambda \left(i\right)=r^{-1}\left(i\right)\left[\begin{array}{c} \Lambda \left(i-1\right)r\left(i\right)+\theta \left(i\right)\theta^{T}\left(i\right)\\ -\theta^{T}\left(i\right) \end{array}\right.\left.\begin{array}{c} -\theta (i)\\ 1 \end{array}\right], \end{array}$$

where θ(i)=Λ(i−1)ξ(i) and $r\left(i\right)=\rho^{i}\zeta 2\sigma^{2}/p\nu \left(i\right)+\varphi^{T}\left(i\right)\varphi \left(i\right)-\theta^{T}\left(i\right)\xi \left(i\right)$. Combining (21) with (26), the coefficients vector α(i) of the weight vector Ω(i) is shown as follows:

(27) $$\begin{array}{cc} \alpha (i)=\Lambda (i)d(i) & =\left[\begin{array}{c} \Lambda \left(i-1\right)+\theta \left(i\right)\theta^{T}\left(i\right)r^{-1}\left(i\right)\\ -\theta^{T}\left(i\right)r^{-1}\left(i\right) \end{array}\right.\left.\begin{array}{c} -\theta \left(i\right)r^{-1}\left(i\right)\\ r^{-1}\left(i\right) \end{array}\right]\left[\begin{array}{c} d(i-1)\\ d(i) \end{array}\right]\\ & =\left[\begin{array}{c} \alpha \left(i-1\right)-\theta \left(i\right)r^{-1}\left(i\right)e\left(i\right)\\ r^{-1}\left(i\right)e\left(i\right) \end{array}\right], \end{array}$$

where $e\left(i\right)=d\left(i\right)-\hat{f}\left(i\right)$ denotes the difference between the desired output d(i) and the system output $\hat{f}\left(i\right)=\xi {\left(i\right)}^{T}\alpha \left(i-1\right)=\sum_{j=1}^{i-1}\alpha_{j}\left(i-1\right)\kappa_{\sigma_{1}}\left(u\left(j\right),u\left(i\right)\right)$. $\alpha_{j}\left(i-1\right)$ is the *j*th element of α(i−1) and all the previous data are the centers. The coefficients α(i−1) and all the previous data should be stored at each iteration. Finally, the RMKRP algorithm is summarized in Algorithm 1. 

**Algorithm 1:** The RMKRP Algorithm.**Initialization**: {u(i),d(i)},i=1,2,… $p,\lambda ,\rho ,\sigma ,\sigma_{1}>0,\zeta \in \left[0,1\right],z\left(1\right)=1-\exp\left(-\frac{d^{2}\left(1\right)}{2\sigma^{2}}\right)$. $H_{1}\left(1\right)=\exp\left(\lambda {\left(z(1)\right)}^{\frac{p}{2}}\right){\left(z(1)\right)}^{\frac{p-2}{2}}\left(1-z\left(1\right)\right)$. $\Lambda \left(1\right)={\left(\zeta \rho 2\sigma^{2}/p/H_{1}\left(1\right)+\kappa \left(u\left(1\right),u\left(1\right)\right)\right)}^{-1},\alpha \left(1\right)=\Lambda \left(1\right)d\left(1\right)$. **Computation**: **While** {u(i),d(i)}(i>1) available **do**
  1) $\xi \left(i\right)={\left[\kappa \left(u\left(1\right),u\left(1\right)\right),...,\kappa \left(u\left(i\right),u\left(i-1\right)\right)\right]}^{T}$  2) $e\left(i\right)=d\left(i\right)-\xi {\left(i\right)}^{T}\alpha \left(i-1\right)$
  3) θ(i)=Λ(i−1)ξ(i)
  4) $z\left(i\right)=1-\exp\left(-\frac{e^{2}\left(i\right)}{2\sigma^{2}}\right)$
  5) $\nu \left(i\right)={\left(\exp\left(\lambda {\left(z(i)\right)}^{\frac{p}{2}}\right){\left(z(i)\right)}^{\frac{p-2}{2}}\left(1-z\left(i\right)\right)\right)}^{-1}$
  6) $r\left(i\right)=\rho^{i}\zeta 2\sigma^{2}/p\nu \left(i\right)+\kappa \left(u\left(i\right),u\left(i\right)\right)-\theta^{T}\left(i\right)\xi \left(i\right)$
  7) $\Lambda \left(i\right)=r^{-1}\left(i\right)\left[\begin{array}{c} \Lambda \left(i-1\right)r\left(i\right)+\theta \left(i\right)\theta^{T}\left(i\right)\\ -\theta^{T}\left(i\right) \end{array}\right.\left.\begin{array}{c} -\theta (i)\\ 1 \end{array}\right]$
  8) $\alpha \left(i\right)=\left[\begin{array}{c} \alpha \left(i-1\right)-\theta \left(i\right)r^{-1}\left(i\right)e\left(i\right)\\ r^{-1}\left(i\right)e\left(i\right) \end{array}\right].$
**end while**


### 3.2. QRMKRP

The RMKRP algorithm generates a linearly growing network owing to the used kernel trick. The online vector quantization (VQ) method [12] has been successfully applied in KAFs to curb its network growth efficiently. Thus, we incorporate the online VQ method into the RMKRP to develop the quantized RMKRP (QRMKRP) algorithm, which is shown as follows.

Suppose that the dictionary C(i) contains *L* vectors at discrete time *i*, i.e., $C\left(i\right)={\left\{C_{k}\left(i\right)\right\}}_{k=1}^{L}$, k∈Id={1,2,…,L}, which means that there are *L* distinctive quantization regions. In the RKHS, the prediction $\hat{f}\left(i\right)$ is therefore expressed as $\hat{f}\left(i\right)=\varphi^{T}\left(C_{k}\left(i\right)\right)\hat{\Omega }$, where $\hat{\Omega }\in F$ is the weight vector in RKHS F. The cost function of QRMKRP based on C(i) is denoted as

(28) $$\begin{array}{c} \sum_{k=1}^{L}\sum_{n=1}^{\left|D_{k}\right|}\rho^{i-k}\frac{1}{\lambda }\exp\left(\lambda {\left(1-\exp\left(-\frac{{\left(d_{kn}\left(i\right)-\varphi^{T}\left(C_{k}\left(i\right)\right)\hat{\Omega }\left(i\right)\right)}^{2}}{2\sigma^{2}}\right)\right)}^{\frac{p}{2}}\right)+\frac{1}{2}\rho^{i}\zeta {∥\hat{\Omega }\left(i\right)∥}^{2}, \end{array}$$

where $\left|D_{k}\right|$ denotes the number of input data those lie in the *k*th quantization region of C(i) and satisfies $\sum_{k\in Id}\left|D_{k}\right|=i$ and $|D_{k}|\geq 1$, and $d_{kn}\left(i\right)$ is the desired output d(i) corresponding to the *n*th element within the *k*th quantization region.

The offline solution to the minimization of (28) can be described by

(29) $$\begin{array}{c} \hat{\Omega }\left(i\right)={\left[\hat{\Phi }\left(i\right)\hat{H}\left(i\right){\hat{\Phi }}^{T}\left(i\right)+\rho^{i}\zeta 2\sigma^{2}/pI\right]}^{-1}\hat{\Phi }\left(i\right)\hat{d}\left(i\right), \end{array}$$

where $\hat{\Phi }\left(i\right)=\left[\varphi \left(C_{1}\left(i\right)\right),\varphi \left(C_{2}\left(i\right)\right),\ldots ,\varphi \left(C_{L}\left(i\right)\right)\right]$ with L≪i elements; $\hat{H}\left(i\right)=\mathrm{diag}\left[\sum_{n=1}^{|D_{1}|}H_{1n}\left(i\right),\sum_{n=1}^{|D_{2}|}H_{2n}\left(i\right),\ldots ,\sum_{n=1}^{|D_{L}|}H_{Ln}\left(i\right)\right]$ denotes a accumulated diagonal matrix; $\hat{d}\left(i\right)=\mathrm{diag}{\left[\sum_{n=1}^{|D_{1}|}H_{1n}\left(i\right)d_{1n}\left(i\right),\sum_{n=1}^{|D_{2}|}H_{2n}\left(i\right)d_{2n}\left(i\right),\ldots ,\sum_{n=1}^{|D_{L}|}H_{Ln}\left(i\right)d_{Ln}\left(i\right)\right]}^{T}$ denotes a accumulated weighted output vector; $H_{kn}\left(i\right)$ denotes $H_{i}\left(i\right)$ corresponding to the *n*th entry of the *k*th quantization region; I denotes an identity matrix with an appropriate dimension. Since (29) has a similar form to (17), we simplify (29) as

(30) $$\begin{array}{c} \hat{\Omega }\left(i\right)=\hat{\Phi }\left(i\right){\left[\hat{H}\left(i\right)\hat{K}\left(i\right)+\rho^{i}\zeta 2\sigma^{2}/pI\right]}^{-1}\hat{d}\left(i\right)=\hat{\Phi }\left(i\right)\hat{Q}\left(i\right)\hat{d}\left(i\right)=\hat{\Phi }\left(i\right)\hat{\alpha }\left(i\right), \end{array}$$

where $\hat{K}\left(i\right)={\hat{\Phi }}^{T}\left(i\right)\hat{\Phi }\left(i\right)$. To obtain the recursive solution to the minimization of (28), we let $\hat{\alpha }\left(i\right)=\hat{Q}\left(i\right)\hat{d}\left(i\right)$ and denote

(31) $$\begin{array}{c} \hat{Q}\left(i\right)={\left[\hat{H}\left(i\right)\hat{K}\left(i\right)+\rho^{i}\zeta 2\sigma^{2}/pI\right]}^{-1}. \end{array}$$

To update $\hat{\Omega }\left(i\right)$ in (30) recursively, two cases are therefore considered.

(1) First, Case: dis(u(i),C(i−1))≤ϵ: In this case, we have C(i)=C(i−1) and $\hat{Q}\left(i\right)=\hat{Q}\left(i-1\right)$, which means the input u(i) is therefore quantized to the $k^{*}$th element of dictionary C(i−1), where $k^{*}$ = $\underset{1\leq k\leq \left|C_{k}\left(i-1\right)\right|}{\arg\min}{∥u(i)-C_{k}\left(i-1\right)∥}^{2}$. The matrix $\hat{H}\left(i\right)$ and the vector $\hat{d}\left(i\right)$ have a similar form to [13]. Here, $\hat{H}\left(i\right)$ and $\hat{d}\left(i\right)$ can be shown as

(32) $$\begin{array}{c} \left\{\begin{array}{c} \hat{H}\left(i\right)=\hat{H}\left(i-1\right)+H_{i}\left(i\right)\tau_{k^{*}}\tau_{k^{*}}^{T}\\ \hat{d}\left(i\right)=\hat{d}\left(i-1\right)+H_{i}\left(i\right)d\left(i\right)\tau_{k^{*}} \end{array}\right., \end{array}$$

where $\tau_{k^{*}}$ is a $\left|C(i-1)\right|$-dimensional column vector whose $k^{*}$th element is 1 and all other elements are 0. Combining (32) into (31), the matrix $\hat{Q}\left(i\right)$ can be expressed as $\hat{Q}\left(i\right)={\left[\hat{Q}{\left(i-1\right)}^{-1}+H_{i}\left(i\right)\tau_{k^{*}}\tau_{k^{*}}^{T}\hat{K}\left(i-1\right)\right]}^{-1}$. By using the matrix inversion lemma [28], we obtain

(33) $$\begin{array}{c} \hat{Q}\left(i\right)=\hat{Q}\left(i-1\right)-\frac{{\hat{Q}}_{k^{*}}\left(i-1\right){\hat{K}}_{k^{*}}^{T}\left(i-1\right)\hat{Q}\left(i-1\right)}{H_{i}^{-1}\left(i\right)+{\hat{K}}_{k^{*}}^{T}\left(i-1\right){\hat{Q}}_{k^{*}}\left(i-1\right)}, \end{array}$$

where ${\hat{Q}}_{k^{*}}\left(i-1\right)$ and ${\hat{K}}_{k^{*}}\left(i-1\right)$ represent the $k^{*}$th columns of the matrices $\hat{Q}\left(i-1\right)$ and $\hat{K}\left(i-1\right)$, respectively. Therefore, $\hat{\alpha }\left(i\right)$ in (30) can be calculated as

(34) $$\begin{array}{c} \hat{\alpha }\left(i\right)=\hat{Q}\left(i\right)\hat{d}\left(i\right)=\hat{\alpha }\left(i-1\right)+\frac{\left(d\left(i\right)-{\hat{K}}_{k^{*}}^{T}\left(i-1\right)\hat{\alpha }\left(i-1\right)\right){\hat{Q}}_{k^{*}}\left(i-1\right)}{H_{i}^{-1}\left(i\right)+{\hat{K}}_{k^{*}}^{T}\left(i-1\right){\hat{Q}}_{k^{*}}\left(i-1\right)}. \end{array}$$

(2) Second Case: dis(u(i),C(i−1))>ϵ: In this case, we have C(i)={C(i−1),u(i)}, $\hat{\Phi }\left(i\right)=\left[\hat{\Phi }\left(i-1\right),\varphi \left(u\left(i\right)\right)\right]$, and we have

(35) $$\begin{array}{c} \hat{H}\left(i\right)=\left[\begin{array}{c} \hat{H}\left(i-1\right)\\ 0^{T} \end{array}\right.\left.\begin{array}{c} 0\\ H_{i}\left(i\right) \end{array}\right],\;\;\hat{K}\left(i\right)=\left[\begin{array}{c} \hat{K}\left(i-1\right)\\ \hat{h}{\left(i\right)}^{T} \end{array}\right.\left.\begin{array}{c} \hat{h}\left(i\right)\\ \kappa_{ii} \end{array}\right], \end{array}$$

where 0 is the null column vector with a compatible dimension; $\hat{h}\left(i\right)=\hat{\Phi }{\left(i-1\right)}^{T}\varphi \left(u\left(i\right)\right)$ and $\kappa_{ii}=\kappa_{\sigma_{1}}\left(u\left(i\right),u\left(i\right)\right)$. Combining (31), (35), $\hat{d}\left(i\right)={\left[\hat{d}{\left(i-1\right)}^{T},H_{i}\left(i\right)d\left(i\right)\right]}^{T}$, and the block matrix inversion identity [28], we obtain

(36) $$\begin{array}{c} \hat{Q}\left(i\right)=\left[\begin{array}{c} \hat{Q}\left(i-1\right)+\hat{r}{\left(i\right)}^{-1}H_{i}\left(i\right){\hat{z}}_{\hat{H}\left(i\right)}\hat{z}{\left(i\right)}^{T}\\ -\hat{r}{\left(i\right)}^{-1}H_{i}\left(i\right)\hat{z}{\left(i\right)}^{T} \end{array}\right.\left.\begin{array}{c} -\hat{r}{\left(i\right)}^{-1}{\hat{z}}_{\hat{H}\left(i\right)}\\ \hat{r}{\left(i\right)}^{-1} \end{array}\right], \end{array}$$

where

(37) $$\begin{array}{c} \left\{\begin{array}{c} {\hat{z}}_{\hat{H}\left(i\right)}=\hat{Q}\left(i-1\right)\hat{H}\left(i-1\right)\hat{h}\left(i\right)\\ \hat{z}\left(i\right)=\hat{Q}{\left(i-1\right)}^{T}\hat{h}\left(i\right)\\ \hat{r}\left(i\right)=\kappa_{\sigma_{1}}\left(u\left(i\right),u\left(i\right)\right)H_{i}\left(i\right)+\rho^{i}\zeta 2\sigma^{2}/p-H_{i}\left(i\right)\hat{h}{\left(i\right)}^{T}{\hat{z}}_{\hat{H}\left(i\right)} \end{array}\right.. \end{array}$$

Furthermore, due to $\hat{d}\left(i\right)={\left[\hat{d}{\left(i-1\right)}^{T},H_{i}\left(i\right)d\left(i\right)\right]}^{T}$, we obtain

(38) $$\begin{array}{c} \hat{\alpha }\left(i\right)=\hat{Q}\left(i\right)\hat{d}\left(i\right)=\left[\begin{array}{c} \hat{\alpha }\left(i-1\right)-\hat{r}{\left(i\right)}^{-1}H_{i}\left(i\right){\hat{z}}_{\hat{H}\left(i\right)}\left(d\left(i\right)-\hat{h}{\left(i\right)}^{T}\hat{\alpha }\left(i-1\right)\right)\\ \hat{r}{\left(i\right)}^{-1}H_{i}\left(i\right)\left(d\left(i\right)-\hat{h}{\left(i\right)}^{T}\hat{\alpha }\left(i-1\right)\right) \end{array}\right]. \end{array}$$

The QRMKRP algorithm is summarized in Algorithm 2, where *L* denotes the dictionary size. 

**Algorithm 2:** The QRMKRP algorithm.**Initialization**: {u(i),d(i)},i=1,2,… $\sigma ,\sigma_{1},p,\lambda >0,L=1,C\left(1\right)=\left\{u\left(1\right)\right\}$, $z\left(1\right)=1-\exp\left(-\frac{d^{2}\left(1\right)}{2\sigma^{2}}\right)$, $H_{1}\left(1\right)=\exp\left(\lambda {\left(z(1)\right)}^{\frac{p}{2}}\right){\left(z(1)\right)}^{\frac{p-2}{2}}\left(1-z\left(1\right)\right),\hat{H}\left(1\right)=\left[H_{1}\left(1\right)\right]$. $\hat{Q}\left(1\right)={\left[\zeta \rho 2\sigma^{2}/p+H_{1}\left(1\right)\kappa_{11}\right]}^{-1},\hat{\alpha }\left(1\right)=\hat{Q}\left(1\right)H_{1}\left(1\right)d\left(1\right)$, ϵ>0,ρ>0,ζ∈[0,1]. **Computation**: **While** {u(i),d(i)}(i>1) available **do**
  1) Compute the distance between u(i) and C(i−1):    dis$\left(u\left(i\right),C\left(i-1\right)\right)=\min_{1\leq k\leq \left|C_{k}\left(i-1\right)\right|}{∥u\left(i\right)-C_{k}\left(i-1\right)∥}^{2}$,    where $k^{*}$ = $\underset{1\leq k\leq \left|C_{k}\left(i-1\right)\right|}{\arg\min}{∥u(i)-C_{k}\left(i-1\right)∥}^{2}$.   2) If dis(u(i),C(i−1))≤ϵ:    Keep the dictionary unchanged: C(i)=C(i−1),L⇐L,    Update $\hat{H}\left(i\right)$ by (32), $\hat{Q}\left(i\right)$ by (33), $\hat{\alpha }\left(i\right)$ by (34).  3) Otherwise:    The dictionary changes: C(i)=[C(i−1),u(i)],L⇐L+1,    Update $\hat{H}\left(i\right)$ by (35), $\hat{Q}\left(i\right)$ by (36), $\hat{\alpha }\left(i\right)$ by (38). **end while**


## 4. Simulation

In this section, to validate the performance of the proposed RMKRP algorithm and its quantized version, two examples, i.e., Mackey–Glass (MG) chaotic time series prediction and nonlinear system identification, are used to validate the performance superiorities of the proposed two algorithms.

In this example, the noise environment considered is the impulsive noise, which is modeled by the combination of two independent noise processes [17], i.e.,

(39) $$v\left(i\right)=\left(1-b\left(i\right)\right)v_{1}\left(i\right)+b\left(i\right)v_{2}\left(i\right),$$

where $v_{1}\left(i\right)$ is an ordinary noise disturbance with small variance and $v_{2}\left(i\right)$ represents large outliers with large variance; b(i) is of binary distribution random process over {0,1} with $Prob\left\{b(i)=1\right\}=c$ and $Prob\left\{b(i)=0\right\}=1-c$ (0≤c≤1 is an occurrence probability). Here, we select c=0.1. The distribution of $v_{1}\left(i\right)$ is considered as a Binary distribution over {0.5,−0.5} with probability mass $Prob\left\{x=0.5\right\}=Prob\left\{x=-0.5\right\}=0.5$. In addition, $v_{2}\left(i\right)$ is modeled by the α-stable process, owing to its heavy-tailed probability density function. The α-stable process is described by the following characteristic function [29]:

(40) $$f\left(t\right)=\exp\left\{j\delta t-\gamma {\left|t\right|}^{\alpha }\left[1+j\beta \mathrm{sgn}(t)S(t,\alpha )\right]\right\},$$

where

(41) $$\begin{array}{c} S\left(t,\alpha \right)=\left\{\begin{array}{c} \tan\frac{\alpha \pi }{2},\mathrm{if}\;\alpha \neq 1\\ \frac{2}{\pi }\log\left|t\right|,\mathrm{if}\;\alpha =1 \end{array}\right., \end{array}$$

with α∈(0,2] being the characteristic factor, β∈[−1,1] being the symmetry parameter, γ>0 being the dispersion parameter, sgn(.) denotes the sign function, $j=\sqrt{-1}$, and −∞<δ<∞ being the location parameter. Generally, a smaller α generates a heavier tail and a smaller γ generates fewer large outliers. The characteristic function denoted as $V_{\alpha -\mathrm{stable}}\left(\alpha ,\beta ,\gamma ,\delta \right)$ is chosen as $V_{\alpha -\mathrm{stable}}\left(0.8,0,0.1,0\right)$ to model the impulse noise in the simulations.

### 4.1. Chaotic Time Series Prediction

The MG chaotic time series is generated from the following differential equation [9]:

(42) $$\frac{dx(t)}{dt}=\frac{\beta x(t-\tau )}{1+x{\left(t-\tau \right)}^{n}}-\gamma x\left(t\right),$$

where β,γ,n>0. Here, we set β=0.2, γ=0.1, and τ=30. The time series is discretized at a sampling period of six seconds. The training set includes a segment of 2000 samples corrupted by the additive noises which are shown in (39), and another 200 samples without noise are used as the testing set. The kernel size $\sigma_{1}$ in the Gaussian kernel is set to 1. The filter length is set at L=7, which means that $\left[x_{t},x_{t-1},\ldots ,x_{t-6}\right]$ is used to predict $x_{t+1}$.

To evaluate the filtering accuracy, the testing mean square error (MSE) is defined as follows:

(43) $$\mathrm{MSE}(\mathrm{dB})=\frac{1}{N}10\mathrm{lo}g_{10}\left(\sum_{i=1}^{N}{\left(d\left(i\right)-\hat{f}\left(i\right)\right)}^{2}\right),$$

where $\hat{f}\left(i\right)$ is the estimate of d(i), and *N* is the length of testing data.

The KLMS [6], KMCC [22], MKRL [26], KRMC [21], and KRLS [8] algorithms are chosen for performance comparison with RMKRP thanks to their excellent filtering performance. The other sparsification algorithms, i.e., the QKLMS [12], QKMCC [30], QMKRL [26], QKRLS [13], and KRMC-NC [21] algorithms are used for performance comparison with QRMKRP owing to their modest space complexities and excellent performance. All simulation results are averaged over 50 independent Monte Carlo runs.

**Figure 2:**
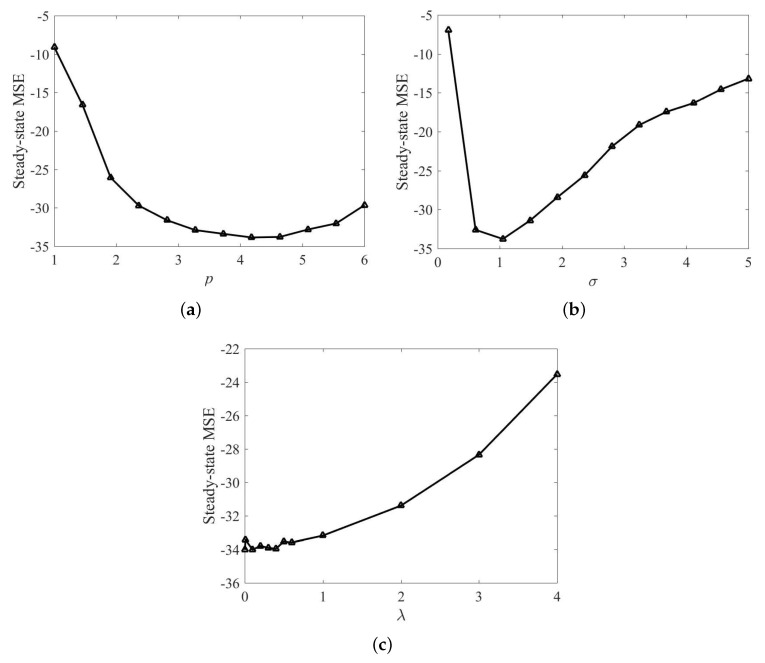
Steady-state MSE of RMKRP with different *p* in MG time series prediction (**a**); steady-state MSE of RMKRP with different σ in MG time series prediction (**b**); steady-state MSE of RMKRP with different λ in MG time series prediction (**c**).

Since power parameter *p*, risk-sensitive parameter λ, and kernel width σ are crucial parameters in the proposed RMKRP and QRMKRP algorithms, the influence of these parameters on the performance is first discussed. In the simulations, we take 12 points evenly in the close interval p∈[1,6] and σ∈[0.17,5], respectively. The influence of *p* on the steady-state performance of RMKRP is shown in Figure 2a, where the steady-state MSEs are obtained as averages over the last 100 iterations. The parameters are set as: *p* is set within [1,6]; risk-sensitive parameter λ in the KRP is set as 1; ζ=0.1 and ρ=1; kernel size σ in the KRP is set as 1. As can be seen from Figure 2a, we have that the filtering accuracy of RMKRP is the highest when p=4 and decreases gradually when *p* is either too small or too large. Then, the influence of σ on the filtering performance of RMKRP with p=4 is shown in Figure 2b, where the steady-state MSEs are obtained as averages over the last 100 iterations. The parameters are set as: risk-sensitive parameter λ is fixed at 1; σ lies in [0.17,5]. From Figure 2b, we see that RMKRP can achieve the highest filtering accuracy when σ is about 1. It is reasonable to note that RMKRP are sensitive to outliers when the kernel width is large, and decreases its ability of error-correction when the kernel width is small. Finally, the influence of λ on the filtering performance of RMKRP with σ=1 and p=4 is shown in Figure 2c, where the steady-state MSEs are obtained as averages over the last 100 iterations. The parameters are set as: the range of λ is selected as λ∈{0.001,0.01,0.1,0.2,0.3,0.4,0.5,0.6,1,2,3,4}. From Figure 2c, we see that λ has a slight influence on the filtering accuracy when λ is small, and a large λ can increase the steady-state MSE obviously. Therefore, from Figure 2, the parameters of RMKRP can be chosen by trials to obtain the best performance in practice. Similarly, the parameters of QRMKRP can be chosen by the same method as that in RMKRP.

**Figure 3:**
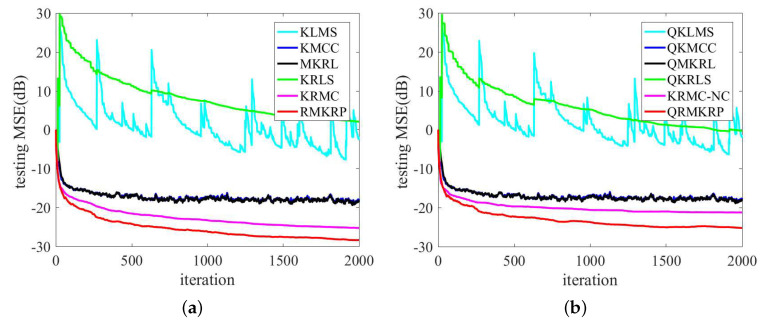
Comparison of the MSEs of KLMS, KMCC, MKRL, KRLS, KRMC, and RMKRP in MG time series prediction (**a**); comparison of the MSEs of QKLMS, QKMCC, QMKRL, QKRLS, KRMC-NC, and QRMKRP in MG time series prediction (**b**).

The performance comparison of QKLMS, QKMCC, QMKRL, KLMS, KMCC, MKRL, KRLS, KRMC, KRMC-NC, and QKRLS is conducted in the same environments as in (39). The parameters of the proposed algorithms are selected by trials to achieve desirable performance, and the parameters of compared algorithms are chosen such that they have almost the same convergence rate. λ=1, p=4, and σ=1 are set for RMKRP; λ=1, p=4, σ=1, and ϵ=0.2 for QRMKRP; η=0.1 for KLMS; η=0.09 and σ=3.5 for KMCC; η=0.09, σ=1, and λ=1 for MKRL; η=0.1 and ϵ=0.2 for QKLMS; η=0.09, ϵ=0.2, and σ=3.5 for QKMCC; η=0.09, ϵ=0.2, σ=1, and λ=1 for QMKRL; ζ=0.1, ρ=1, and σ=3.5 for KRMC; the novelty criterion thresholds $\delta_{1}=0.15$, $\delta_{2}=0.1$, ζ=0.1, ρ=1, and σ=3.5 for KRMC-NC; ζ=0.1 for KRLS; ζ=0.1 and ϵ=0.2 for QKRLS. Figure 3 shows the compared MSEs of RMKRP, QRMKRP, and the compared algorithms. As can be seen from Figure 3, RMKRP achieves a better filtering accuracy than KRLS, KRMC, KLMS, KMCC, and MKRL. QRMKRP achieves a better steady-state testing MSE than the sparsification algorithms including QKRLS, KRMC-NC, QKLMS, QKMCC, and QMKRL. We also see from Figure 3 that the proposed algorithms provide good robustness to impulsive noises. For detailed comparison, the dictionary size, consumed time, and steady-state MSEs in Figure 3 are shown in Table 1. Note that the steady-state MSEs of KLMS, QKLMS, KRLS, and QKRLS are not shown in Table 1 since they cannot converge in such impulsive noise environment. From Table 1, we see that RMKRP has similar consumed time to KRLS and KRMC but provides better filtering accuracy. In addition, QRMKRP provides the highest filtering accuracy in all the compared sparsification algorithms and approaches the filtering accuracy of RMKRP with a significantly lower network size.

### 4.2. Nonlinear System Identification

To further validate the performance superiorities of the proposed RMKRP and QRMKRP algorithms, the nonlinear system identification is considered. Here, the nonlinear system is of the following form [31].

(44) $$\begin{array}{cc} s(t) & =s\left(t-1\right)\left(0.8-0.5\exp\left(-s^{2}\left(t-1\right)\right)\right)-\left(0.3+0.9\exp\left(-s^{2}\left(t-1\right)\right)\right)s\left(t-2\right)+0.1\sin\left(s\left(t-1\right)\pi \right), \end{array}$$

where s(t) denotes the output at discrete time *t* with the initial s(−1)=0.1 and s(−2)=0.1. The two previous outputs $u\left(k\right)={\left[s\left(t-1\right),s\left(t-2\right)\right]}^{T}$ are utilized as the input to estimate the current output s(t). The training set includes a segment of 2000 samples corrupted by the additive noises shown in (39), and another 200 samples without noise are used as the testing set. The kernel width $\sigma_{1}$ is set to 1 for the Gaussian function. All simulation results are averaged over 50 independent Monte Carlo runs.

**Figure 4:**
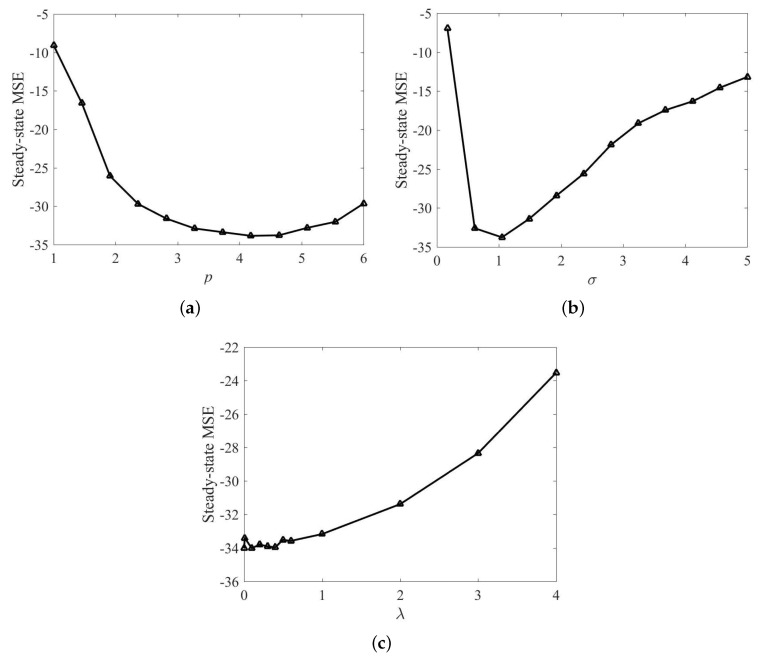
Steady-state MSE of RMKRP with different *p* in nonlinear system identification (**a**); steady-state MSE of RMKRP with different σ in nonlinear system identification (**b**); steady-state MSE of RMKRP with different λ in nonlinear system identification (**c**).

Similar to MG chaotic time series prediction, the influence of power parameter *p*, risk-sensitive parameter λ, and kernel width σ on the performance of RMKRP is also discussed in nonlinear system identification. The influence of *p* on the steady-state performance of RMKRP is shown in Figure 4a, where the steady-state MSEs are obtained as averages over the last 100 iterations. The parameters are set as: *p* is set within [1,6]; λ is set as 0.1; ζ=0.1 and ρ=1; kernel size σ in the KRP is set as 1. The influence of σ on the filtering performance of RMKRP is shown in Figure 4b, where risk-sensitive parameter λ is fixed at 0.1; σ lies in [0.17,5]; *p* is set as 4. The influence of λ on the filtering performance of RMKRP is shown in Figure 4c, where the range of λ is selected as λ∈{0.001,0.01,0.1,0.2,0.3,0.4,0.5,0.6,1,2,3,4}; σ is set as 1; *p* is set as 4. As can be seen from Figure 4, we can obtain the same conclusions as those in Figure 2.

**Figure 5:**
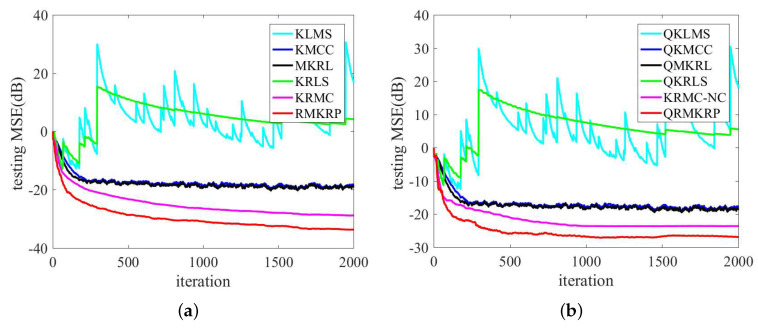
Comparison of the MSEs of KLMS, KMCC, MKRL, KRLS, KRMC, and RMKRP in nonlinear system identification (**a**); comparison of the MSEs of QKLMS, QKMCC, QMKRL, QKRLS, KRMC-NC, and QRMKRP nonlinear system identification (**b**).

We compare the filtering performance of QKLMS, QKMCC, QMKRL, KLMS, KMCC, MKRL, KRLS, KRMC, KRMC-NC, and QKRLS in the same environments as in (39). The parameters of the proposed algorithms are selected by trials to achieve desirable performance, and the parameters of compared algorithms are chosen such that they have almost the same convergence rate. λ=0.1, p=4, and σ=1 are set for RMKRP; λ=0.1, p=4, σ=1, and ϵ=0.2 for QRMKRP; η=0.1 for KLMS; η=0.09 and σ=3.5 for KMCC; η=0.09, σ=1, and λ=2 for MKRL; η=0.1 and ϵ=0.2 for QKLMS; η=0.09, ϵ=0.2, and σ=3.5 for QKMCC; η=0.09, ϵ=0.2, σ=1, and λ=2 for QMKRL; ζ=0.1, ρ=1, and σ=3.5 for KRMC; the novelty criterion thresholds $\delta_{1}=0.01$, $\delta_{2}=0.1$, ζ=0.1, ρ=1, and σ=3.5 for KRMC-NC; ζ=0.1 for KRLS; ζ=0.1 and ϵ=0.2 for QKRLS. Figure 5 shows the compared MSEs of RMKRP, QRMKRP, and the compared algorithms. For detailed comparison, the dictionary size, consumed time, and steady-state MSEs in Figure 5 are also shown in Table 2, where the steady-state MSEs of KLMS, QKLMS, KRLS, and QKRLS are not shown since they cannot converge in such impulsive noise environments. From Figure 5 and Table 2, we can obtain the same conclusions as those in Figure 3 and Table 1.

## 5. Conclusions

In this paper, the kernel risk-sensitive mean *p*-power error (KRP) criterion is proposed by constructing mean *p*-power error (MPE) into kernel risk-sensitive loss (KRL) in RKHS, and some basic properties are presented. The KRP criterion with power parameter *p* is more flexible than KRL to handle the signal corrupted by impulsive noises. Two kernel recursive adaptive algorithms are derived to obtain desirable filtering accuracy under the minimum KRP (MKRP) criterion, i.e., the recursive minimum KRP (RMKRP) and quantized RMKRP (QRMKRP) algorithms. The RMKRP can achieve higher accuracy but with almost identical computational complexity as that of the KRLS and KRMC. The vector quantization method is introduced into RMKRP, thus generating QRMKRP, and QRMKRP can effectively reduce network size while maintaining the filtering accuracy. Simulations conducted in Mackey–Glass (MG) chaotic time series prediction and nonlinear system identification under impulsive noises illustrate the superiorities of RMKRP and QRMKRP from the aspects of robustness and filtering accuracy.

## Figures and Tables

**Table 1:** Simulation results of QKLMS, QKMCC, QMKRL, QKRLS, KRMC-NC, KLMS, KMCC, MKRL, KRLS, KRMC, RMKRP, and QRMKRP in MG time series prediction.

Algorithms	Size	Time (s)	MSE (dB)
KLMS [6]	2000	30.9501 s	N/A
QKLMS [12]	28	2.1011 s	N/A
KRLS [8]	2000	58.5358 s	N/A
QKRLS [13]	28	2.3374 s	N/A
KMCC [22]	2000	30.8285 s	−18.5063
QKMCC [30]	28	2.0995 s	−17.8707
MKRL [26]	2000	30.9117 s	−18.7312
QMKRL [26]	28	2.1063 s	−18.1037
KRMC [21]	2000	58.1229 s	−25.1618
KRMC-NC [21]	462	2.8045 s	−21.5183
QRMKRP	28	2.3443 s	−24.9326
RMKRP	2000	58.2196 s	−28.1802

**Table 2:** Simulation results of QKLMS, QKMCC, QMKRL, QKRLS, KRMC-NC, KLMS, KMCC, MKRL, KRLS, KRMC, RMKRP, and QRMKRP in nonlinear system identification.

Algorithms	Size	Time (s)	MSE (dB)
KLMS [6]	2000	21.2447 s	N/A
QKLMS [12]	14	1.7284 s	N/A
KRLS [8]	2000	48.6055 s	N/A
QKRLS [13]	14	1.9643 s	N/A
KMCC [22]	2000	21.1328 s	−19.233
QKMCC [30]	14	1.763 s	−17.9723
MKRL [26]	2000	21.0313 s	−19.5390
QMKRL [26]	14	1.7243 s	−18.5748
KRMC [21]	2000	48.7601 s	−28.7583
KRMC-NC [21]	496	2.6874 s	−23.671
QRMKRP	14	1.9681 s	−27.3128
RMKRP	2000	48.6101 s	−34.0790
